# Genome-wide identification of transcriptional enhancers during human placental development and association with function, differentiation, and disease^†^

**DOI:** 10.1093/biolre/ioad119

**Published:** 2023-09-23

**Authors:** David M Owen, Minjung Kwon, Xuan Huang, Anusha Nagari, Tulip Nandu, W Lee Kraus

**Affiliations:** Laboratory of Signaling and Gene Regulation, Cecil H. and Ida Green Center for Reproductive Biology Sciences, University of Texas Southwestern Medical Center, Dallas, TX, USA; Division of Basic Research, Department of Obstetrics and Gynecology, University of Texas Southwestern Medical Center, Dallas, TX, USA; Division of General Obstetrics and Gynecology, Department of Obstetrics and Gynecology, University of Texas Southwestern Medical Center, Dallas, TX, USA; Laboratory of Signaling and Gene Regulation, Cecil H. and Ida Green Center for Reproductive Biology Sciences, University of Texas Southwestern Medical Center, Dallas, TX, USA; Division of Basic Research, Department of Obstetrics and Gynecology, University of Texas Southwestern Medical Center, Dallas, TX, USA; Laboratory of Signaling and Gene Regulation, Cecil H. and Ida Green Center for Reproductive Biology Sciences, University of Texas Southwestern Medical Center, Dallas, TX, USA; Division of Basic Research, Department of Obstetrics and Gynecology, University of Texas Southwestern Medical Center, Dallas, TX, USA; Laboratory of Signaling and Gene Regulation, Cecil H. and Ida Green Center for Reproductive Biology Sciences, University of Texas Southwestern Medical Center, Dallas, TX, USA; Division of Basic Research, Department of Obstetrics and Gynecology, University of Texas Southwestern Medical Center, Dallas, TX, USA; Laboratory of Signaling and Gene Regulation, Cecil H. and Ida Green Center for Reproductive Biology Sciences, University of Texas Southwestern Medical Center, Dallas, TX, USA; Division of Basic Research, Department of Obstetrics and Gynecology, University of Texas Southwestern Medical Center, Dallas, TX, USA; Laboratory of Signaling and Gene Regulation, Cecil H. and Ida Green Center for Reproductive Biology Sciences, University of Texas Southwestern Medical Center, Dallas, TX, USA; Division of Basic Research, Department of Obstetrics and Gynecology, University of Texas Southwestern Medical Center, Dallas, TX, USA

**Keywords:** placenta, trophoblast, stem cell, gene expression, transcription factor, genomics, RNA-sequencing (RNA-seq), chromatin immunoprecipitation-sequencing (ChIP-seq)

## Abstract

The placenta is a dynamic organ that must perform a remarkable variety of functions during its relatively short existence in order to support a developing fetus. These functions include nutrient delivery, gas exchange, waste removal, hormone production, and immune barrier protection. Proper placenta development and function are critical for healthy pregnancy outcomes, but the underlying genomic regulatory events that control this process remain largely unknown. We hypothesized that mapping sites of transcriptional enhancer activity and associated changes in gene expression across gestation in human placenta tissue would identify genomic loci and predicted transcription factor activity related to critical placenta functions. We used a suite of genomic assays [i.e., RNA-sequencing (RNA-seq), Precision run-on-sequencing (PRO-seq), and Chromatin immunoprecipitation-sequencing (ChIP-seq)] and computational pipelines to identify a set of >20 000 enhancers that are active at various time points in gestation. Changes in the activity of these enhancers correlate with changes in gene expression. In addition, some of these enhancers encode risk for adverse pregnancy outcomes. We further show that integrating enhancer activity, transcription factor motif analysis, and transcription factor expression can identify distinct sets of transcription factors predicted to be more active either in early pregnancy or at term. Knockdown of selected identified transcription factors in a trophoblast stem cell culture model altered the expression of key placental marker genes. These observations provide a framework for future mechanistic studies of individual enhancer–transcription factor–target gene interactions and have the potential to inform genetic risk prediction for adverse pregnancy outcomes.

## Teaser

Mapping sites of transcriptional enhancer activity and associated gene expression in human placenta across gestation identifies genomic loci and predicted transcription factor activity related to critical placenta functions.

## Graphical Abstract

**Graphical Abstract:**
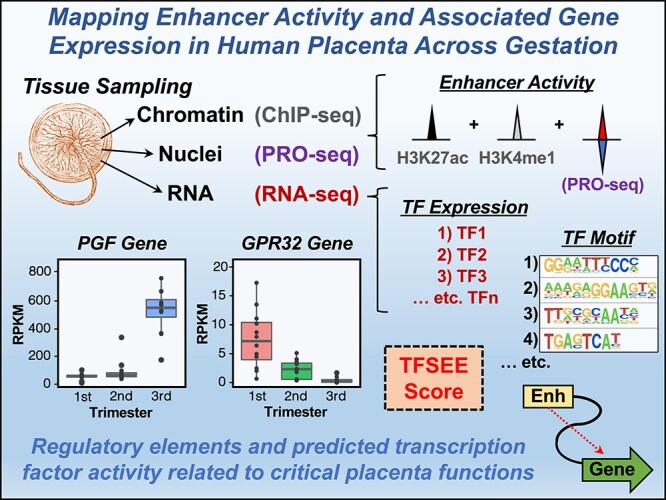

## Introduction

The placenta is a dynamic organ that performs a remarkable variety of functions, including nutrient delivery, gas exchange, waste removal, hormone production, and immune barrier functions [1, 2]. Many of these functions take place within the floating villi of the placenta where maternal blood comes into direct contact with the syncytiotrophoblast (ST) [3, 4] The ST is formed as underlying cytotrophoblasts differentiate and fuse to form this multinucleated layer that increases in surface area over the course of pregnancy [5]. As villi develop into their highly branched mature forms, the relative proportion of cytotrophoblasts and their trophoblast stem cell (TSC) precursors decreases [3, 4]. Altered villous maturation has been associated both with preterm birth and stillbirth [6, 7]. Furthermore, release of soluble vasoactive factors from the placenta plays a role in the development of preeclampsia [8, 9].

While proper placenta development and function is critical for healthy pregnancy outcomes, the underlying genomic regulatory events that control this process are only beginning to be understood [10–12]. In this regard, a number of studies using microarrays [13–16], bulk RNA-seq [17–19], and single cell RNA-seq (scRNA-seq) [20–27] have examined aspects of how gene expression in the placenta changes over time in normal or pathologic pregnancies [10, 12, 28]. A number of studies have focused on differences in gene expression in placenta tissue in response to disease [12, 22, 27–33], while others have used genomic profiling to define distinct cell types in the placenta [10, 11, 21, 33–37]. Fewer studies, however, have examined how diverse genomic features [e.g., transcription, histone modification, DNA methylation, transcription factor (TF) binding, chromatin accessibility, and chromatin interactions] change over the course of normal development. This is partly due to the limited accessibility of placenta tissue from early pregnancy samples, which results in an incomplete picture of gene regulation, and its associated epigenomic events such as enhancer formation. Nonetheless, current genomics technologies can facilitate the analysis of these genomic features in available tissues, including those features that define transcriptional enhancers [11, 38–40].

Enhancers are genomic regulatory elements that function as nucleation sites for the binding of sequence-specific TFs and the formation of regulatory complexes that can communicate with the promoters of target genes [41]. Enhancers are characterized by common molecular features such as (1) an open or accessible chromatin environment; (2) enrichment of a common set of histone modifications, such as histone H3 lysine 4 (H3K4) monomethylation and histone H3 lysine 27 (H3K27) acetylation; (3) binding of TFs, coregulators, and chromatin remodeling enzymes; and (4) looping to target gene promoters [41, 42]. Enhancers are also actively transcribed, producing enhancer RNAs (eRNAs) [42]. Differential accumulation of these molecular features defines distinct classes of enhancers that specify distinct gene regulatory mechanisms and biological outcomes. Enhancer transcription and eRNAs are thought to function by (1) promoting the recruitment of TFs and coregulators and regulating their activities; (2) facilitating RNA polymerase II (RNAPII) pause-release to promote transcription elongation; and (3) driving enhancer–promoter looping [42]. The NIH ENCODE and Roadmap epigenomics projects have annotated enhancer elements from a limited set of placenta samples on the basis of chromatin accessibility (Deoxyribonuclease-sequencing; DNase-seq) or histone modifications.

Our lab and others have shown that enhancer transcription and the production of eRNAs are the most specific marker of active enhancers [43–45]. The sites of enhancer transcription can be identified by mapping short unstable eRNA transcripts from nuclear run-on assays, such as precision run-on sequencing (PRO-seq) data. This technique uses biotinylated ribonucleotides in an in vitro transcription reaction from isolated nuclei followed by pull-down and sequencing to identify sites of active transcription. This approach, however, has not yet been applied to the study of enhancers in human placenta tissue. In this work, we used a multi-faceted approach combining genomic and computational analyses to define changes in the enhancer landscape and gene expression across gestation in normal pregnancy, using samples from all three trimesters. While machine learning approaches have been applied to predict new placenta-specific enhancers from existing genomic data [46], our work approximately doubles the number of available samples with experimentally determined enhancer annotations and does so using enhancer transcription, a more stringent marker of enhancer activity.

## Materials and methods

### Source of placenta tissue

Placenta tissue was collected from patients who provided informed consent; it was obtained in a de-identified manner from a tissue bank at UT Southwestern under an approved IRB protocol (term samples) or from Advanced Bioscience Resources, Alameda, CA (early pregnancy samples). The latter was obtained prior to January 1, 2019. Tissue was from otherwise normal, uncomplicated pregnancies collected at the time of scheduled cesarean delivery or elective pregnancy termination. Samples were taken from multiple sites for each placenta, using four quadrant biopsies from term placenta and approximating this strategy to the extent permitted from early pregnancy tissues. The maternal decidual surface and fetal amnionic surfaces were excluded or removed, with sampling performed from the region of floating villi in the interior of the placenta. Tissue was rinsed in PBS, flash-frozen, and stored at −80°C until processed. Multiple tissue samples were pooled from an individual placenta for extraction of RNA, nuclei, or chromatin in order to achieve more representative sampling across multiple locations in a single placenta.

### RNA extraction and RNA-seq library construction

Frozen tissue was pulverized using a liquid nitrogen–chilled hammer mill and then homogenized in Trizol. Following chloroform-mediated phase separation, the total RNA was purified from the aqueous phase using RNeasy columns (Qiagen, 74104) according to the manufacturer’s protocol. The RNA concentration was quantified using a NanoDrop, and the quality was assessed by RNA ScreenTape (Agilent). The total RNA was stored at −80°C until processed. Following standards defined by the Genome-Tissue Expression (GTEx) project [47], only samples with an RNA integrity number (RIN) score > 6 were used for library construction. Ten micrograms of total RNA were used as input for polyA RNA-seq library construction as previously described [48].

### Nuclei extraction and PRO-seq library construction

Frozen tissue was pulverized using a liquid nitrogen–chilled hammer mill, homogenized in ice-cold Swelling Buffer [10 mM Tris–HCl pH 8, 2 mM magnesium acetate, 3 mM calcium chloride, 0.25 mM DTT, and 1x cOmplete protease inhibitor cocktail (Roche, 11697498001)], and passed through a 40 micron filter. The nuclei were released from the pulverized tissue in Lysis Buffer (Swelling Buffer containing 10 mM NaCl, 300 mM sucrose, and 0.5% NP-40), washed three times in Lysis Buffer, and flash-frozen in Nuclei Freezing Buffer (50 mM Tris–HCl pH 8.3, 5 mM magnesium chloride, 0.1 mM EDTA, 40% glycerol) in aliquots of 5 × 10^6^ nuclei. Nuclear run-on was performed at 37°C for 5 min using biotin-labeled CTP [49]. PRO-seq libraries were constructed following the protocol of Mahat and colleagues [49].

### Chromatin preparation and ChIP-seq library construction

Frozen tissue was pulverized using a liquid nitrogen–chilled hammer mill, crosslinked with 1% formaldehyde in a volume of 10 mL, quenched by addition of 5 mL of 2.5 M glycine for 5 min at 4°C, washed generously in ice-cold PBS, and homogenized in Farnham Lysis Buffer (5 mM PIPES pH 8, 85 mM KCl, 0.5% NP-40, 1 mM DTT, 1x cOmplete protease inhibitor cocktail), as described [50]. The nuclei were collected by brief centrifugation and resuspended in SDS Lysis Buffer (50 mM Tris–HCl pH 7.9, 1% SDS, 10 mM EDTA, 1 mM DTT, 1x cOmplete protease inhibitor cocktail) by pipetting and incubating on ice for 10 min. The chromatin was sheared to ~200 bp DNA fragments by sonication using a Bioruptor sonicator (Diagenode) for 24–28 cycles of 30 s on and 30 s off. Fragment size was verified by agarose gel electrophoresis before quantification of protein concentrations using a BCA protein assay kit (Pierce, 23225). One hundred micrograms of soluble chromatin was precleared with Protein A Dynabeads (Invitrogen, 10001D) before incubation overnight with 2.5 μg of antibody: H3K4me1 (Abcam, ab8895) or H3K27ac (Abcam, ab4729). ChIP-seq libraries were constructed as described previously [51].

### Next-generation sequencing

The genomic libraries with compatible barcodes were pooled and sequenced on the Illumina NextSeq 500 in a 75 bp single-end format. The libraries were sequenced to an average depth as follows: RNA-seq ~52 M reads per sample; PRO-seq: ~73 M reads per sample; and ChIP-seq ~32 M reads per sample.

### Analysis of RNA-seq data

#### Quality check and preprocessing RNA-seq libraries

The raw data were subjected to QC analyses using the FastQC tool [52]. The reads were then mapped to the human genome (hg38) using the spliced reader aligner TopHat version.2.0.13 [53]. Uniquely mappable reads were converted into bigWig files using BEDTools (version 2.17.0) for visualization in the Integrative Genomics Viewer (version 2.9.4). Transcriptome assembly was performed using cufflinks (version 2.2.172) [54] with default parameters using the aligned reads. The transcripts were merged into distinct, non-overlapping sets using cuffmerge, followed by cuffdiff to call the differentially regulated transcripts.

#### Data normalization

Using cuffdiff, FPKMs and fragment counts were scaled via the median of the geometric means of fragment counts across all libraries, as described by Anders and Huber [55]. The resulting normalized FPKM values were used to compare the genes regulated in the three trimesters with linked enhancer transcription via statistical methods implemented in R, such as Pearson correlation analysis, linear regression of FPKM versus gestational age in weeks, and differences in mean FPKM value between groups using *t*-tests with correction using the Benjamini–Hochberg procedure. The Benjamini–Hochberg procedure was used to adjust *p*-values for multiple hypothesis testing. The normalized FPKM values were also used in subsequent downstream analyses, including differential expression by trimester, correlation with gestational age, and integrated analysis with ChIP-seq and PRO-seq data as described below.

#### Gene Ontology enrichment analysis

Pathways associated with genes that increase or decrease in the placenta across pregnancy were identified using Gene Ontology (GO) annotations [56] for GO biological process gene sets and the PANTHER enrichment analysis tool [57].

### Analysis of ChIP-seq data

The reads were trimmed using Cutadapt (version 1.9.1) [58] and mapped to the hg38 reference genome using Bowtie (version 1.0.0) [59]. Output files were converted into BED files using SAMTools (version 0.1.19) [60] and BEDTools (version 2.17.0) [61]. The aligned reads were used to measure library complexity using BEDTools (v 2.17.0) [61] and met minimum ENCODE data quality standards [62]. Using aligned reads as input, we used MACS (version 2.1.0) software [63] to call peaks from ChIP-seq data using the default *p*-value and input condition as a control. Uniquely mapped reads were visualized on the UCSC genome browser as bigWig files generated using BEDTools [61].

### Analysis of PRO-seq data and prediction of enhancers using dREG

#### Quality check and preprocessing PRO-seq libraries using Proseq2.0

The quality of the data was confirmed using FastQC software [52]. The PRO-seq libraries were analyzed using Proseq2.0 pipeline [64] and aligned to human reference genome hg38 with ~72% average alignment percentage. The aligned bam files were converted into bigwig format using deeptools (v2.3.5) [65] and bedGraphToBigWig [66] program to visualize in the UCSC genome browser [67].

#### Identification of enhancers using dREG

The unnormalized bigwig files generated from Proseq2.0 were used to predict enhancers/transcription regulatory elements (TREs) using the dREG package on the dREG computational gateway [68]. We then built a universe of transcripts by merging the dREG peak calls from individual samples across each gestational age and stratifying the boundaries to remove overlaps/redundancies occurring from the union of all the dREG peak calls using BEDtools merge (v2.17.0) [61]. Next, we calculated the RPKM of the union of TREs or peak calls by collecting the read counts from bedtools multicov [61] and filtering the results to identify a subset of short intergenic transcripts >5 kb away from the 5′ or 3′ ends of annotated genes using bedtools intersect [61]. The final universe of expressed distal TREs (*n* = 20 502) combined from all samples was determined from the PRO-seq data using an RPKM cutoff ≥2 in at least 1 out of the 36 samples across the gestational age.

#### Identification of TF motifs using MEME

We performed de novo motif analyses on a 1 kb region (±500 bp) surrounding the TRE summit for expressed TREs in each sample using the command-line version of MEME [69]. The following parameters were used for motif prediction: (1) zero or one occurrence per sequence (− mod zoops); (2) number of motifs (−nmotifs 15); (3) minimum, maximum width of the motif (− minw 8, −maxw 15); and (4) search for motif in given strand and reverse complement strand (− revcomp). The predicted motifs from MEME were matched to known motifs using Tomtom [70] using the Jaspar 2018 database [71].

### Predicting trimester-specific TFs using Total Functional Score of Enhancer Elements

We used the Total Functional Score of Enhancer Elements (TFSEE) algorithm as described previously [72, 73] to combine PRO-seq, RNA-seq, and ChIP-seq data with TF motif information to predict the TFs that drive the formation of active enhancers in placenta across gestation. In order to use the TFSEE pipeline, we obtained the following information for the final universe of expressed distal TREs (*n* = 20 502) in 15 samples that had PRO-seq, RNA-seq, and ChIP-seq data available: (1) enhancer transcription values using PRO-seq; (2) histone modification enrichment values at the enhancers using ChIP-seq (H3K4me1 and H3K27ac); (3) motif search results; and (4) TF expression values using RNA-seq. The TFSEE algorithm was then applied to determine the enhancer activity by normalizing the enhancer transcription and histone modification enrichment and then integrating with the motif predictions and TF expression to calculate the final TFSEE score [72, 73]. To identify trimester-specific TFs, we performed hierarchical clustering by Pearson correlation and the average linkage method. The rank order of the TFs that were enriched between the clades comprising trimesters I and II versus trimester III was calculated as described [72, 73].

### Enhancer single-nucleotide polymorphism analysis

The NHGRI-EBI genome-wide association study (GWAS) catalog was downloaded on 9/1/2020 and contained 130 789 single-nucleotide polymorphisms (SNPs) associated with any outcome by GWAS. The bedtools intersect function [61] was used to count the number of SNPs overlapping either the annotated genes (GENCODE transcripts) or the placenta enhancers identified in this study. SNP density was calculated by dividing the entire SNP catalog by the total genome size or the resulting overlapping SNPs by the size of these regions in base pairs to generate SNPs per million base pairs.

### Data visualization

Data from the different sequencing methodologies were visualized and compared in browser track representations using the Integrative Genomics Viewer (IGV; Broad Institute). After data analysis using the relevant statistical tools or packages in R, visual representations in the form of boxplots, bar graphs, and dot plots were generated in R using the ggplot2 package [74]. Heatmaps were generated using Java Tree View [75] or the heatmap function in R.

### Trophoblast stem cell culture and differentiation

#### Growth and differentiation

A TSC line developed by Okae et al. [76] was cultured under conditions that they optimized. For TSC propagation, the cells were grown on collagen-coated plates in basal medium [DMEM/F12 supplemented with 0.003% BSA, 1x ITS-X (Wako Chemicals, 094–06761), 0.1 mM β-mercaptoethanol] supplemented with 0.05 μg/mL EGF (Sigma, E9644), 0.002% FBS, 1.5 μg/mL ascorbic acid, 0.0125% valproic acid (HDAC inhibitor; Wako Chemicals, 227–01071), 5 μM Y27632 (ROCK inhibitor; Stemcell Technologies, 72,304), 2 μM CHIR99021 (Wnt activator; Wako Chemicals, 034-23103), 0.5 μM A83-01 (TGF-β inhibitor; Wako Chemicals, 035-24113), and 1 μM SB431542 (TGF-β inhibitor; Wako Chemicals, 031-24291). Differentiation toward the ST lineage was performed in 2D culture using basal medium supplemented with 4% KnockOut Serum Replacement (ThermoFisher, 10828010), 2.5 μM Y27632, and 2 μM forskolin (Sigma, F6886).

#### Marker analyses, morphologic visualization, and confirmations

Gene markers of ST differentiation (*CGB*, *ERVW-1*, *CYP19A1*, *GCM1*) were assessed following 1–5 days of culture in differentiation medium using Taqman probes as described below. For visualization of morphology changes, TSCs were plated in collagen-coated chamber slides and grown under TSC propagation or ST differentiation conditions as described above. Membranes were stained with Di-8-ANNEPPS (ThermoFisher, D3167) and nuclei visualized with DAPI. Fresh cell stocks were regularly replenished from the original stocks every few months, verified for cell type identity using the GenePrint 24 system (Promega, B1870), and confirmed as mycoplasma-free every 3 months using a commercial testing kit.

### RNAi-mediated TF depletion in TSCs

We used two approaches to knockdown TFs in the TSCs and SCs: (1) lentiviral-mediated delivery of shRNA constructs (to knockdown *ZBTB7C* and *SNAI2*) and (2) lipofectamine-mediated delivery of siRNAs (to knockdown *GCM1*).

#### Lentiviral-mediated delivery of shRNA constructs

For the former, we used shRNA constructs targeting human *SNAI2* mRNA (TRCN0000284362) or human *ZBTB7C* mRNA (TRCN0000235012), or a control shRNA (SHC002), all of which were purchased from Sigma. We generated lentiviruses by transfection of the constructs described above, together with (i) an expression vector for the VSV-G envelope protein (pCMV-VSV-G, Addgene plasmid no. 8454); (ii) an expression vector for GAG-Pol-Rev (psPAX2, Addgene plasmid no. 12260); and (iii) a vector to aid with translation initiation (pAdVAntage, Promega) into 293 T cells using Lipofectamine 3000 Reagent (Invitrogen, L3000015) according to the manufacturer’s protocol. The resulting viruses were collected in the culture medium, concentrated by using a Lenti-X concentrator (Clontech, 631231), and used to infect TSC cells seeded at a density of 2 × 10^5^. Stably transduced cells were selected with puromycin (Sigma, P9620; 2.5 μg/mL) in cell culture medium.

#### Lipofectamine-mediated delivery of siRNAs

In a 6-well plate, 0.5 × 10^6^ TSCs were seeded. After attachment, the cells were transfected using Lipofectamine RNAiMAX reagent (Invitrogen,13778150) according to the manufacturer’s instruction. Briefly, 30 pmole of siRNA in 150 μL optiMEM and 9 μL of RNAiMAX in 150 μL optiMEM were combined, incubated for 5 min, and dispensed into a single well. Seventy-two hours after transfection, the cells were collected for analysis.

### Reverse transcription-quantitative PCR

Total RNA was extracted from cultured cells using an RNAeasy kit (Qiagen, 74104), quantified by using a NanoDrop; mRNA expression was analyzed using the following approaches.

#### Standard reverse transcription-quantitative PCR

Complementary DNA pools were prepared from TSCs using the RNeasy kit (Qiagen), followed by reverse transcription using MMLV reverse transcriptase (Promega, M150B) with oligo(dT) primers (Sigma-Aldrich). The cDNA was treated with 3 units of RNase H (Ambion) for 30 min at 37°C and then analyzed by qPCR using the primer sets listed below and a LightCycler 480 real-time PCR thermocycler (Roche) for 45 cycles. The delta–delta Ct (2^–∆∆Ct^) method was used to analyze comparative fold changes in the gene expression level [77].

MAFK Forward 5’-CTGCGCTCCAAGTACGAGGCG-3’

MAFK Reverse 5’-TCGGTGGACTTGACGATGGTGA-3’

MAFB Forward 5’-AGACGCCTACAAGGTCAAGTGC-3’

MAFB Reverse 5’-CGACTCACAGAAAGAACTCGGG-3’

SNAI2 Forward 5’-TTTTCCAGACCCTGGTTGCTT-3’

SNAI2 Reverse 5’-GAGCCCTCAGATTTGACCTGT-3’

IRF1 Forward 5’-GAGGAGGTGAAAGACCAGAGCA-3’

IRF1 Reverse 5’-TAGCATCTCGGCTGGACTTCGA-3’

ZBTB7C Forward 5’-GGAGAAGCCATACATGTGCACC-3’

ZBTB7C Reverse 5’-ACGAACTTGGCGTTGCAGTGGA-3’

TFAP2A Forward 5’-GACCTCTCGATCCACTCCTTAC-3’

TFAP2A Reverse 5’-GAGACGGCATTGCTGTTGGACT-3’

#### Taqman assay

RNA was reverse-transcribed using the High Capacity cDNA Reverse Transcription kit (ThermoFisher, 4368814). Quantitative PCR was performed using Taqman probes listed below (ThermoFisher), Taqman Fast Advanced Master Mix (ThermoFisher, 4444554), and 10 ng cDNA template in a 384-well format. Amplification and detection were performed using a LightCycler 480 real-time PCR thermocycler (Roche), and fold changes in expression were calculated using the delta–delta Ct (2^–∆∆Ct^) method [77].

Taqman *CGB3* assay Hs00361224_gH

Taqman *CYP19A1* assay Hs00903411_m1

Taqman *ERVW-1* assay Hs00205893_m1

Taqman *GCM1* assay Hs00172692_m1

Taqman *HLA-G* assay Hs00365950_g1

### Western blotting

Nuclear extracts were prepared from either undifferentiated or differentiated trophoblast as described previously [78]. The protein concentrations of the extracts were quantified using a Bradford assay (Biorad, 500-0006). Ten micrograms of total protein were run on 12% acrylamide SDS-PAGE gels and transferred to nitrocellulose membranes by wet transfer for 120 min. Blocking, incubation with antibodies, and washing of blots were done in TBST buffers, with blocking for 1 h in 3% milk, incubation with primary antibody overnight, and HRP-conjugated secondary antibody (Bethyl Labs; 1:6 000 dilution) for 1 h. Signals were visualized by chemiluminescence with SuperSignal Pico Plus regent (ThermoFisher, 1863096) and imaged on the ChemiDoc system (BioRad).

### Genomic data set availability

The following new data sets generated for this study are available from the NCBI’s Gene Expression Omnibus (GEO) database (http://www.ncbi.nlm.nih.gov/geo/) using accession number GSE222035.

## Results

### Collection and processing of normal placenta samples for genomic analyses

**Figure 1:**
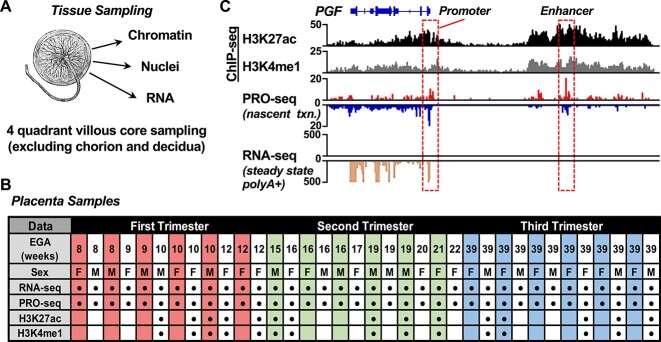
Multidimensional placenta genomic profiling across gestation enables enhancer annotation linked to gene expression. (A) Schematic representation of placenta tissue sample processing for genomic assays. We collected first-, second-, and third-trimester placenta tissue from otherwise normal, uncomplicated pregnancies at the time of elective pregnancy termination or scheduled cesarean delivery. Samples were taken from multiple sites for each placenta and multiple tissue fragments were pooled on a per-sample basis for extraction of RNA, nuclei, or chromatin to prepare genomic libraries for deep sequencing assays (RNA-seq, ChIP-seq, PRO-seq). (B) Gestational age, sex, and libraries constructed from the placenta tissues collected. Sex was determined based on Y chromosome markers from RNA-seq. EGA, estimated gestational age in weeks. Exact gestational ages for the third-trimester placentas are as follows: P08 - 39w1d; P09 - 39w1d; P10 - 39w0d; P11 - 39w2d; P12 - 39w0d; P13 - 39w0d; P21 - 39w0d; P32 - 39w0d; P47 - 39w1d; P48 - 39w3d; P49 - 39w0d; P50 - 39w0d, where “w” is weeks and “d” is days. Due to the nature of the collections for the first and second trimester placental samples, we do not have data more granular than gestational age in weeks. (C) Genome browser tracks showing ChIP-seq (H3K27ac, H3K4me1), PRO-seq, and RNA-seq from a 39-week placenta sample showing the promoter region and a putative enhancer (both marked with hatched boxes) near the placenta growth factor (*PGF*) gene (the gene schematic is shown).

In order to identify changes in gene expression and associated changes in the enhancer landscape during normal pregnancy, we prepared multidimensional genomic libraries from a set of placenta samples representing normal development across all three trimesters of pregnancy. The 12 samples collected per trimester were balanced for fetal sex (Figure 1A and B). Samples were obtained with informed consent from patients with otherwise uncomplicated pregnancies undergoing scheduled cesarean delivery (not in labor) or from patients undergoing elective termination (without known fetal anomalies). As reflected in the sample characteristics (Figure 1B), our intention to model placenta development across “normal” pregnancy resulted in a gap of accessible tissue from the time of viability to term.

We isolated RNA, chromatin, or nuclei (Figure 1A) from bulk placenta tissue samples due to the input requirements for the nuclear run-on reactions used in our enhancer mapping strategy. This represents a tradeoff between the ability to assign findings to specific cell types (as with single-cell methodologies) and the types of assays and information that can be obtained with limiting amounts of starting material. As many critical placenta functions occur at the maternal–fetal interface of the ST surface, we focused on the floating villi in the interior of the placenta, which make up the greatest portion of the placenta by volume and surface area. Although this sampling generates a composite signature from multiple cell types (primarily trophoblasts, fetal vessels, and placental macrophages), complexity was reduced by excluding the fetal surface and associated membranes, as well as the decidual surface with associated maternal tissue, extravillous trophoblasts, and decidual immune cell populations.

### Determining gene expression and enhancer landscapes in normal placenta

To determine changes in gene expression in placental villi, RNA-seq and precision run-on sequencing [49] libraries were constructed from all 36 samples and sequenced to an average depth of ~52 and ~ 73 M million mapped reads, respectively. We identified transcription units from the PRO-seq data using dREG [79]. We then defined active enhancer regions for all samples based on enhancer transcription from PRO-seq, which we and others have shown to be a highly specific marker of active enhancers [72]. To isolate likely enhancers from other transcription units, the transcripts were filtered for location in intergenic regions >5 kb away from the transcription start sites (TSSs) of known genes as annotated by GENCODE [80]. An RPKM threshold cutoff of >2 in at least 1 sample was applied to reduce background noise and false positives. This filtering resulted in a universe of 20 502 enhancers across all samples, with between ~2 500 and ~ 10 000 enhancers identified in each of the 36 samples (Supplementary Table S1). We further characterized the predicted enhancers using chromatin immunoprecipitation (ChIP-seq) for H3K27ac and H3K4me1 in a representative subset of five samples per trimester. The multiple layers of genomic information facilitated the identification of enhancers located near genes related to placenta function, such as the gene encoding placenta growth factor (*PGF*) (Figure 1C).

### Placenta gene expression is dynamic across pregnancy

**Figure 2:**
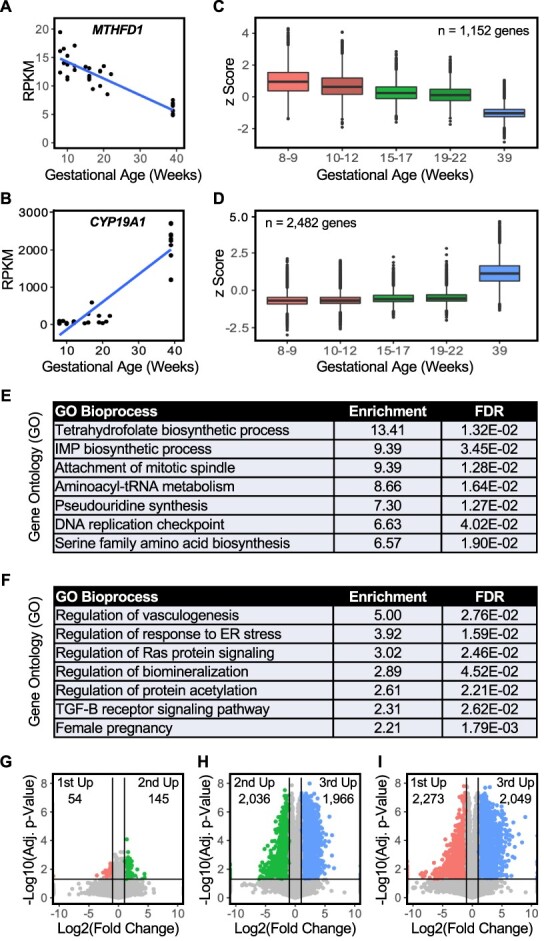
Gene expression is dynamic across pregnancy. RNA-seq analysis of placenta gene expression across gestation. Differential expression analyses identified 3634 genes whose expression correlated with gestation age in weeks. (A–D) Analysis of individual genes whose expression decreases (*MTHFD1*; A) or increases (*CYP19A1*; B) across gestation shown as scatter plots with linear regression. Box plots (C and D) show trends for all genes where expression is negatively correlated (C) or positively correlated (D) with increasing gestational age. (E and F) Gene ontology analysis for sets of genes whose expression decreases (E) or increases (F) across gestation. (G–I) Pairwise analyses of gene expression between trimesters identifies more significant gene expression differences for the third trimestercompared to the first or second trimesters than between the first and second trimesters.

Next, we sought to explore changes in gene expression across gestation using two different approaches. In the first approach, we identified genes whose expression (in RPKM) was either positively or negatively correlated with gestational age in weeks, using Pearson *R* > 0.7 or < −0.7, and a significant *p*-value for the correlation after Bonferroni correction for multiple hypothesis testing (*p* < 2.17 × 10^−6^ for 23 000 tested associations) (Supplementary Table S2). This analysis resulted in 1152 genes with decreased expression across gestation and 2482 genes that increased across gestation. Examples of such genes are shown in Figure 2A and B (*MTHFD1* and *CYP19A1*). *MTHFD1* encodes an enzyme in the DNA synthesis pathway downstream from folate, whereas *CYP19A1* encodes the aromatase enzyme involved in estrogen synthesis in STs. To visualize overall trends in gene expression that increased or decreased across gestation, the expression of individual genes within a sample were z-score-normalized, binned into five groups of increasing gestational age, and plotted as box plots (Figure 2C and D).

We performed GO analyses for genes correlated with gestational age (Figure 2E and F; Supplementary Table S3). For the set of genes more highly expressed early in pregnancy, there was enrichment for terms associated with DNA synthesis, DNA replication, and cell division. This period of cell proliferation in early pregnancy may be associated with a pool of self-renewing TSCs that decreases in proportion or activity later in pregnancy. In support of this idea, we observed that the gene encoding telomerase (*TERT*) was expressed in 10 out of the 12 first-trimester samples (0.1–0.4 RPKM) and absent in later samples. Interestingly, for the set of genes more highly expressed later in pregnancy, GO analysis identified terms related to vasculogenesis and TGFβ signaling (Figure 2F). Vasculogenesis is required for the dramatic expansion of the fetal capillary network in mature villi [81]. Moreover, inhibition of TGFβ is critical for maintaining the TSC phenotype in culture, and thus, signaling through this pathway may be important for differentiation. Indeed, TGFβ has been implicated in both syncytium formation and regulating invasiveness of extravillous trophoblasts [82].

We also performed pairwise comparisons of samples grouped by trimester as an alternate approach for determining gene expression changes across pregnancy. Figure 2G–I shows genes whose expression exhibits greater than a 2-fold difference (vertical lines) between trimesters based on a significant *t*-test *p*-value with Benjamini–Hochberg correction (see also Supplementary Table S4). These data again show that the most substantial differences in gene expression are between term and early pregnancy.

Finally, we explored the possibility that the relative proportion of cell types, as well as changes in gene expression within a given cell type, could change across gestation and that both processes might contribute to the signal obtained from bulk tissue analysis. We used gene signatures from single-cell RNA-seq obtained from The Human Protein Atlas database enriched in cytotrophoblasts (420 genes), STs (790 genes), and extravillous trophoblasts (680 genes). By comparing the average expression of these gene sets in our RNA-seq datasets from the gestational age epochs defined in Figure 2C and D, we observed that the extravillous trophoblast and cytotrophoblast signals do not significantly change between gestational age groups, while the ST signal is significantly increased in the third trimester, as would be expected (Supplementary Fig. S1).

### Linking placenta gene expression to enhancer activity across pregnancy

**Figure 3:**
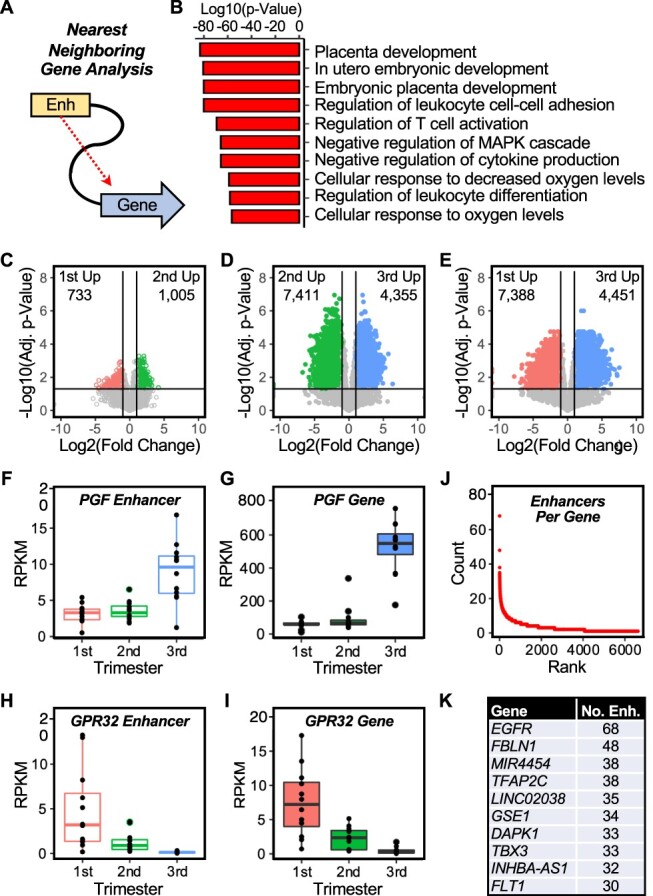
Enhancer activity is dynamic across pregnancy, and associates with genes related to placenta function and disease. Analysis of the transcription of putative placenta enhancers defined by PRO-seq across gestation. This analysis identified a total of 20 502 enhancers. (A) Schematic analysis of the “nearest neighboring gene” analysis used to associate enhancers with target genes. (B) Gene ontology analysis of the nearest neighboring genes to the putative placenta enhancers defined by PRO-seq. (C–E) Pairwise analyses of enhancer transcription between trimesters identifies more significant enhancer transcription differences for the third trimester compared to the first and second trimesters. (F–I) Examples of genes whose enhancer transcription (F and H) correlates with nearest neighboring gene expression (G and I) across gestation. *PGF* (F and G), both enhancer transcription and nearest neighboring gene expression increase; *GPR32* (H and I), both enhancer transcription and nearest neighboring gene expression decrease. (J and K) Enhancers per gene analysis. (J) Rank order plot of the number of putative placenta enhancers per differentially expressed gene across gestation. Most genes had only one or two potential enhancers identified from nearest neighboring gene analysis. (I) Genes with the highest potential for enhancer-mediated regulation include TFs and cell signaling molecules associated with trophoblast differentiation (*TFAP2C*, *EGFR*) and disease (*FLT1*).

After demonstrating that gene expression in the placenta is dynamic and reflects known placenta functions, we sought to link placenta gene expression with the enhancer landscape. We associated the 20 502 enhancers that we defined by their PRO-seq transcription signature with the nearest neighboring gene (Figure 3A). To assess whether the genes identified in this analysis are the same as those identified in Figure 2 using only RNA-seq, we determined the relationship between nearest neighboring genes and the enriched enhancers determined by both approaches. Although only a minority of the genes overlap, in all cases where overlap exists, the number of genes overlapping (i.e., identified independently by both differential RNA-seq and nearest neighbor enhancer activity) is significant by the hypergeometric distribution test (i.e., when compared to the probability of overlap for randomly selected gene lists in sets of the same size) (Supplementary Table S5 and Supplementary Figure S2). We then determined gene ontologies for the associated genes using the Genomic Regions Enrichment of Annotations Tool (GREAT) [83]. As shown in Figure 3B, the top GO bioprocesses for the nearest neighboring genes were terms related to placenta development. In a more detailed analysis, we overlapped the 20 502 enhancers in our data set with the 961 227 cis-regulatory elements (cCREs) annotated by ENCODE and found that our data set still contains 3 550 unique enhancer regions. GREAT analysis for this subset, like the entire set (Figure 3B), shows GO terms related to placenta development (Supplementary Table S6). This suggests that our transcription-based enhancer analysis efficiently identifies genomic regions relevant to the control of placenta-specific gene expression.

Next, we determined if the enhancer landscape, like the gene expression landscape, is dynamic across gestation. We examined changes in enhancer transcription in pairwise comparisons between trimesters similar to the analyses we did for gene expression above. As we observed for gene expression, the biggest differences in the active enhancer landscape were between early and late pregnancy (Figure 3C–E; Supplementary Table S7). When we focused on genes with placenta-enriched expression relative to other tissues, we observed dynamic changes in enhancer activity that correlated with changes in nearest neighboring gene expression by trimester. This was evident for the genes encoding placenta growth factor (*PGF*; Figure 3F and G) and GPR32 (*GPR32*, a G-protein coupled receptor enriched in trophoblasts; Figure 3H and I) (for additional genes, see Supplementary Tables 1–3 and 7).

Enhancers act in cell type– and tissue type–specific manners to fine tune the expression of genes important for those systems. We hypothesized that genes particularly important for placenta function may be linked to multiple enhancer elements. We determined the number of enhancers per gene for the ~6 000 genes linked to a nearest neighboring enhancer from above. While the majority of linked genes have only one or two nearby enhancers, a small number of genes are linked to dozens of enhancers (Figure 3J). Notably, the gene with the most linked enhancers, encoding epidermal growth factor receptor (*EGFR*) (Figure 3K), is important for trophoblast function and is altered in cases of intrauterine growth restriction [84]. Likewise, *FLT1* (encoding vascular endothelial growth factor receptor 1), another gene linked to many enhancers, is dysregulated in preeclampsia [85]. These findings suggest the possibility that enhancer dysfunction could play a role in adverse pregnancy outcomes*.*

### Placental enhancers and disease associations

**Figure 4:**
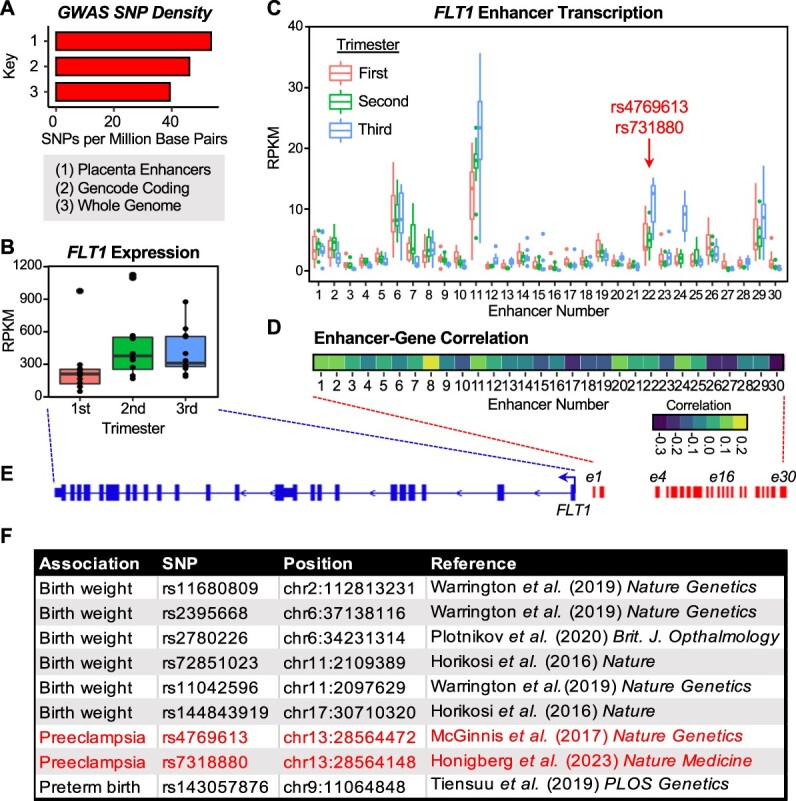
Placenta enhancers associate with risk for disease and pregnancy outcomes. (A) Single-nucleotide polymorphisms associated with disease in genome-wide association studies (GWASs) are enriched in enhancers relative to genomic background. Single-nucleotide polymorphisms in placenta enhancers defined by PRO-seq versus all GENCODE coding genes and the whole genome. (B) Expression across gestation of *Fms related receptor tyrosine kinase 1* (*FLT1*), a gene that is linked to enhancers and is dysregulated in preeclampsia, from RNA-seq data. (C) Transcription levels of 30 putative enhancers located near the *FLT1* gene determined by PRO-seq. Two previously reported SNPs associated with preeclampsia (rs4769613 [87, 88]; rs7318880 [89]) map to a putative *FLT1* enhancer (e22). (D) Correlation of the transcription of putative *FLT1* enhancers with *FLT1* expression. A scale bar is shown. (E) Schematic diagram of the *FLT1* gene and 30 putative enhancers (e1 through e30). (F) Additional SNPs associated with pregnancy outcomes, which map to enhancers identified in this study.

Genome-wide association studies have identified many thousands of SNPs associated with disease outcomes or other population variants. About half of these SNPs map to genomic regions outside of annotated transcripts, often corresponding to enhancer regions. To explore the possibility that enhancer loci identified in our analyses could be linked to disease phenotypes, we mapped disease-associated SNP density within enhancer elements relative to genes (GENCODE transcripts) using SNPs from a publicly available catalog [86]. We found that GWAS SNPs are enriched in the placental enhancers that we identified (Figure 4A), including one located upstream of *FLT1*, a gene whose expression increases in the second and third trimesters versus the first trimester (Figure 4B). Although GWAS associations are understudied in pregnancy relative to other disease states, the most robust GWAS SNP association for preeclampsia, rs4769613 (replicated in two studies [87, 88]), maps to the *FLT1* enhancer region (Figure 4C–E; enhancer number 22). Interestingly, activity at the rs4769613-containing enhancer (determined by transcription levels), like *FLT1* expression varies with gestational age (Figure 4B and C) and is positively correlated (though weakly) with *FLT1* gene expression (Figure 4D; Supplementary Table S8). Similar results were observed with another preeclampsia-associated SNP, rs7318880 (Figure 4C–E; also in enhancer number 22) [89], providing independent identification of additional genetic variation at this enhancer associated with placenta-related disease and strengthening the biological plausibility of this enhancer serving a functional role in the placenta.

To further explore the link between genetic variation in enhancer regions and placenta function, we overlapped the location of eQTLs for gene expression in placenta tissue reported by Peng et al. [90] with the enhancers that we defined. Of the 1216 eQTLs from extragenic regions of the genome reported in that study, 80 of these map to enhancer regions we identified. These 80 enhancers and the cognate genes linked to the eQTLs are listed in Supplementary Table S9. Additional GWAS SNPs associated with pregnancy outcomes mapping within our enhancer data set are summarized in Figure 4F [87, 91–94]. Of note, these associations are between the fetal genome and the outcome of interest. Maternal GWASs linked to pregnancy outcomes via the placenta are more difficult to detect since only half of the SNPs in the placenta would be expected to derive from the maternal genome.

### Predicting the transcription factors that promote enhancer formation and drive placental biology

**Figure 5:**
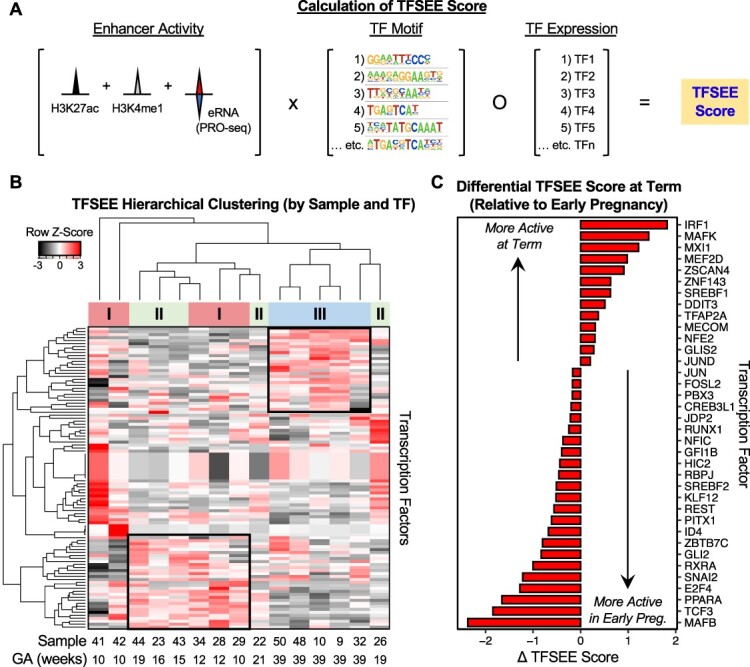
Integrated genomics predicts TF activity at enhancers across gestation. (A) Schematic for determination of TFSEE scores from multidimensional genomic datasets. For each enhancer, the top 20 conserved motifs were identified and linked to TF binding motifs with an associated *p*-value using MEME [69]. Transcription factor expression was multiplied by this matrix to yield a TFSEE score for each TF identified on a per sample basis. (B) Unsupervised hierarchical clustering identifies TFs where the TFSEE score is increased at term (top-right black box) or early pregnancy (bottom-left black box). (C) Differential TFSEE scores for individual TFs at term relative to early pregnancy. We identified TFs with statistically different TFSEE scores between early and late pregnancy (*t*-test, *p* < 0.05). We ranked the TFs by differential TFSEE score (TFSEE_late_ − TFSEE_early_) to identify those most likely to function at different times in pregnancy (“active in early pregnancy” and “active at term” as indicated).

Enhancer formation and activity is due to the binding of TFs at these loci. We sought to integrate enhancer activity (enhancer transcription by PRO-seq; H3K27ac and H3K4me1 enrichment by ChIP-seq), TF motif sequence information, and TF expression (by RNA-seq) to identify the TFs most likely mediating enhancer formation and biological outcomes in the placenta (Figure 5A). To accomplish this, we used a computational model developed in our lab, the TFSEE [72, 73]. Using the subset of 15 samples (5 per trimester) for which the required input data were available (PRO-seq, H3K27ac and H3K4me1 ChIP-seq, and RNA-seq), we determined enhancer activity scores for each enhancer in each sample (Figure 5A).

For each enhancer, the top 20 conserved motifs were identified and linked to TF binding motifs with an associated *p*-value using MEME [69]. Finally, TF expression was multiplied by this matrix to yield a TFSEE score for each TF identified on a per-sample basis. Unsupervised hierarchical clustering of TFSEE scores largely grouped early pregnancy (first and second trimester) samples together, distinct from term pregnancy samples (Figure 5B). Given this empirical clustering pattern, we then identified TFs with statistically different TFSEE scores between early and late pregnancy (*t*-test, *p* < 0.05). We ranked the TFs by the differential TFSEE score (TFSEE_late_ – TFSEE_early_) to identify those most likely to function at specific times during pregnancy, including MAFB, SNAI2, and ZBTB7C active in early pregnancy, as well as IRF1, MAFK, and TFAP2A active at term (Figure 5C; Supplementary Table S10). Interestingly, pairs of TFs were identified on opposite ends of the TFSEE differential (MAFK vs. MAFB; SREBF1 vs. SREBF2), suggesting possible competition between related TFs for binding to common enhancer elements at different times during gestation.

### Exploring the expression, activity, and function of TFSEE–identified transcription factors in trophoblast stem cells

**Figure 6:**
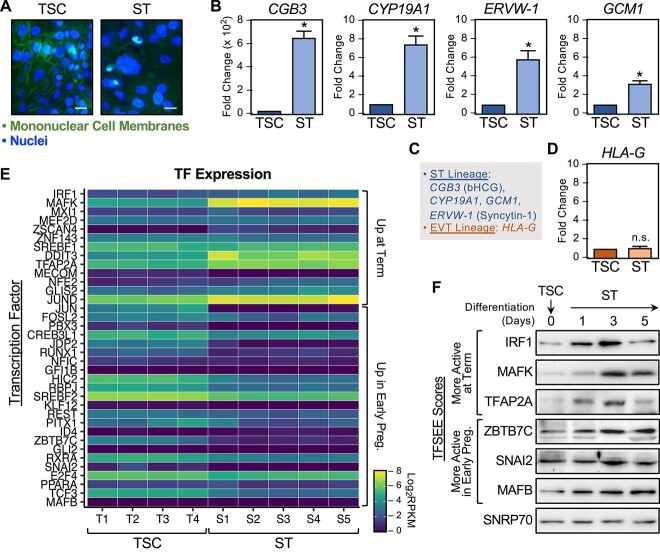
Expression of TFSEE-identified TFs from placenta in TSCs and STs. In vitro differentiation of TSCs to STs using a culture system developed by Okae and colleagues [76]. (A) Verification of the differentiation of TSCs to STs based on cell morphology. Staining for mononuclear cell membranes and DNA as indicated. Scale bar = 5 μm. (B–D) Verification of the differentiation of TSCs to STs based on marker gene expression by RT-qPCR. (B) Expression of ST-specific marker genes (*Chorionic gonadotropin subunit beta 3*, *CG3B*; *Cytochrome P450 family 19 subfamily A member 1*, *CYP19A1*; *Endogenous retrovirus group W member 1*, *ERVW-1*; and *Glial cells missing transcription factor 1*, *GCM1*). (C) Key showing the marker genes representing the ST lineage and the extravillous trophoblast lineage. (D) Minimal expression of an extravillous trophoblast marker gene (*Major histocompatibility complex class I G*, *HLA-G*) in STs. Significance was determined by Student *t*-test; * = *p* < 0.05, n.s. = not significant. (E) Heatmap TF expression for multiple cell lines and replicates under TSC propagation conditions (two replicates each of two cell lines) or ST differentiation conditions (two or three replicates each of two cell lines). The TFs are grouped based on their expression (“up in early pregnancy” and “up at term” as indicated). (F) Expression of key TF proteins during a 5-day time course of differentiation from TSCs to STs determined by Western blotting of nuclear extracts. The results from the TFSEE analyses are shown for comparison (“active in early pregnancy” and “active at term” as indicated).

To explore the expression, activity, and function of selected TFSEE-identified TFs in a defined cell system, we used the TSC culture protocol developed by Okae and colleagues [76] under stem cell propagation conditions [e.g., in the presence of EGF, ascorbic acid, valproic acid, ROCK inhibitor (Y27632), Wnt activator (CHIR99021), ALK-5 inhibitor (A83–01), and TGFβ inhibitor (SB431542)]. In some cases, we also stimulated differentiation of the TSCs toward the ST lineage (e.g., in differentiation medium including Y27632 and forskolin for 1–5 days, replacing the medium every 2 days). We verified the differentiation of TSCs to STs by (1) morphology (loss of mononuclear cell membranes staining due to cell fusion) (Figure 6A), (2) appropriate expression of ST-specific marker genes (including *CG3B*, *CYP19A1*, *ERVW-1*, and *GCM1*) (Figure 6B and C), and (3) minimal expression of an extravillous trophoblast (EVT) marker gene (*HLA-G*) (Figure 6C and D).

In further analyses, we examined the expression of the TFSEE-identified TFs in the published RNA-seq data sets from the Okae et al.’s paper [76]. The heatmap in Figure 6E shows the expression of selected TFs from that study for multiple cell lines and replicates under TSC propagation conditions (two replicates each of two cell lines) or ST differentiation conditions (two or three replicates each of two cell lines), focusing on the TFs highlighted in Figure 5C. The TFSEE scores from our analyses (Figure 5C) and the TF mRNA expression from the RNA-seq data (Figure 6E) trended similarly in some cases (i.e., early pregnancy vs. term; TSC vs. STs), but overall, the relationships were modest. This observation is unsurprising since TF mRNA expression is only one of a number of parameters that drives TFSEE score (Figure 5A).

Next, we examined the expression (protein and mRNA) of the selected TFs noted above (i.e., MAFB, SNAI2, and ZBTB7C active in early pregnancy, as well as IRF1, MAFK, and TFAP2A active at term) during a time course of differentiation from TSCs to STs by Western blotting and reverse transcription-quantitative PCR (RT-qPCR), respectively. The nuclear levels of IRF1, MAFK, and TFAP2A protein were low in TSCs, maximal by Day 3 of differentiation, and then tapered off by Day 5 (Figure 6F). In contrast, the nuclear levels MAFB, SNAI2, and ZBTB7C protein were elevated in TSCs and continued to increase during differentiation, but less dramatically than for IRF1, MAFK, and TFAP2A (Figure 6F). The expression levels of the *IRF1*, *MAFK*, and *TFAP2A* mRNAs from RNA-seq and RT-qPCR followed the expression levels of the proteins, but the expression levels of the *MAFB*, *SNAI2*, and *ZBTB7C* mRNAs exhibited some variability and disconnect between the mRNA and protein levels for some TFs (Supplementary Figure S3). Nonetheless, the protein and mRNA expression data tracked with and segregated the “more active in early pregnancy” (i.e., MAFB, SNAI2, and ZBTB7C) and “more active at term” (i.e., IRF1, MAFK, and TFAP2A) TFSEE-defined TFs, as expected.

**Figure 7:**
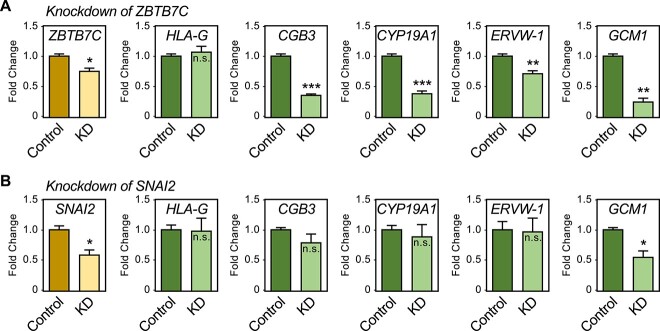
Activity and function of TFSEE-identified TFs in TSCs. Perturbation-response assays in undifferentiated TSCs, with shRNA-mediated knockdown of mRNAs encoding TFSEE-identified TFs, ZBTB7C and SNAI2. The expression of mRNAs encoding the knocked down TFs, as well as a set of EVT (*HLA-G*) and ST (*CGB3*, *CYP19A1*, *ERVW-1*, and *GCM1*) markers was assessed by RT-qPCR. Significance was determined by Student *t*-test; * = *p* < 0.05, ** = *p* < 0.01, and *** = *p* < 0.001, n.s. = not significant. (A) Effects of knockdown of *ZBTB7C*. (B) Effects of knockdown of *SNAI2*.

Finally, we explored the activity and function of two TFs, ZBTB7C and SNAI2, which are predicted by TFSEE to be more active in early pregnancy, but are expressed both in TSCs and throughout the time course of differentiation (Figure 6F). To do so, we used an RNAi-mediated knockdown perturbation-response assay in undifferentiated TSCs. Knockdown of *ZBTB7C* reduced the expression of a number of ST marker mRNAs, including *GCM1*, but had no effect on the expression of *HLA-G* mRNA, a marker of EVTs (Figure 7A). In contrast, knockdown of *SNAI2* had little effect on most of the same markers, but it did cause a reduction in the levels of *GCM1* mRNA (Figure 7A). As a control, we knocked down *GCM1* and assayed for the expression of marker genes. We observed a significant reduction in the levels of *CGB3* and *ERVW-1* mRNA, markers of the ST lineage (Supplementary Figure S4). Our results indicate that TFSEE can identify functionally relevant TFs in placenta whose perturbation can alter the expression of marker genes in TSCs.

## Discussion

In this work, we have provided a detailed analysis of the normal human placental transcriptome—both for steady-state RNA and active transcription—across gestation. In addition, we have used a computational algorithm called TFSEE to define the TFs that are likely to drive key aspects of placental biology during each trimester. Specifically, we have shown that (1) gene transcription and mRNA expression in the placenta across gestation is dynamic; (2) enhancer activity defined by enhancer transcription is also dynamic; (3) active enhancers in the placenta are located near genes relevant to placenta function; (4) placental enhancers are subject to genetic variation associated with disease; (5) integrated genomic data analysis using TFSEE identifies TFs that are predicted to change in activity across gestation, which may be missed by solely focusing on TF expression; (6) the expression of TFs predicted from TFSEE analysis are altered during the differentiation of TSCs to STs; and (7) knockdown of selected identified TFs in a TSC culture model altered the expression of key placental marker genes. These observations provide a framework for future mechanistic studies of individual enhancer–TF–target gene interactions and have the potential to inform genetic risk prediction for adverse pregnancy outcomes.

### Connecting enhancer activity and gene expression to placenta function through gene ontology

Our analyses demonstrate how enhancer activity and gene expression can be used to understand placenta biology using the functions of individual genes or the ontologies of sets of genes. This can provide information that connects specific TFs to specific functions at different stages of placental development. For example, we showed that *MTHFD1*, a gene encoding an enzyme involved in the pathway of DNA synthesis downstream from folate, and thymidylate synthase (not shown) are more highly expressed in early pregnancy, consistent with the sensitivity of early trophoblasts to methotrexate [95] and reflected in the gene ontology analysis for pathways enriched in early pregnancy (Figure 2A, C, and E). In contrast, *CYP19A1* (Figure 2B), a gene encoding the aromatase enzyme involved in estrogen synthesis in STs, increases across gestation, consistent with the ~100 fold increase in estrogen levels across pregnancy due to production in the placenta (Figure 2B and D).

Also associated with increased gestational age are genes related to vasculogenesis (Figure 2F), consistent with the dramatic expansion of the fetal capillary network in mature villi. The “female pregnancy” term in Figure 2F represents the production of known placenta-derived gene products, such as the pregnancy-specific glycoprotein (PSG) family members and hormones including CRH, which peaks in the third trimester. There were also several terms related to endoplasmic reticulum (ER) stress and the unfolded protein response. Placental ER stress has been associated with intrauterine growth restriction [96]. These findings in normal tissue at term could reflect the increased stress an aging placenta experiences, as well as an increased risk for stillbirth when pregnancy proceeds post-term and fetal demands outstrip the ability of the placenta to respond.

Finally, we found evidence for a rapidly dividing population of cells with active enhancers and the expression of marker genes associated with stem cells in early pregnancy relative to term (Figure 5C). These results suggest that genomics analyses from a mixed cell population can still yield insights about a limited cell type in that population. In this regard, we confirmed that TFs identified by our combinatorial genomics approach from placenta tissue were also altered in a cell culture model of TSC differentiation. These included TFs active in early pregnancy (i.e., MAFB, SNAI2, and ZBTB7C) and at term (i.e., IRF1, MAFK, and TFAP2A) (Figure 6A), which may define subsets of TFs that control TSC propagation or differentiation into STs, respectively.

### Connecting enhancer activity and gene expression to specific transcription factors

In gene-specific analyses, we explored the activity and function of selected TFSEE-identified TFs in TSCs grown in culture [76]. These included ZBTB7C and SNAI2, TFs that are predicted by TFSEE to be more active in early pregnancy, but are expressed both in TSCs and throughout the time course of differentiation (Figure 6F). Our results using an RNAi-mediated knockdown perturbation-response assay in undifferentiated TSCs indicate that TFSEE can identify functionally relevant TFs in placenta whose perturbation can alter the expression of marker genes in TSCs.

### Connecting enhancer activity to disease through GWAS SNPs

Linking enhancer activity and gene expression through correlation analysis can generate testable hypothesis about the regulation of particular genes important to placenta function. Many genomic studies rely on combining genomic features from data sets generated in different samples in order to generate regulatory predictions. The data sets of enhancer annotations generated in this study, including both enhancer activity and gene expression from the same sample, along with histone modification data from a significant subset of those samples, will be a rich resource for mining associations between regulatory regions and predicted target genes.

With regard to understanding disease, we demonstrated that our enhancer annotations can provide context for the functional understanding of GWAS SNPs as they emerge. Such annotations serve to narrow the sequence space of interest when generating hypotheses about the relationship between genetic variation and disease outcomes. They could also suggest functional regions in linkage disequilibrium with lead SNPs, which might explain the SNPs that are responsible for the effects of the association. These associations could be tested in future studies using functional genomics approaches targeting both the enhancer via emerging functionalized dCas9 reagents, and the TFs predicted by motif analysis and expression patterns in knock out/knock down approaches.

### Limitations of the study

While this study represents an advance over previous studies with respect to the numbers and types of samples analyzed, as well as the suite of genomic assays used, it is not without limitations. Regarding the biological systems that we used, one obvious limitation is the absence of placenta collections from gestational age from 22 to 39 weeks for practical, technical, legal, and ethical reasons. While this limited our ability to fully examine gene expression across gestation, we were able to describe general trends by gestational age or trimester within the limits of available samples, as well as connect enhancer activity to gene expression in this particular biological system.

Another limitation is the tissue sampling that we used, especially with the larger, term placentas, which may result in the sampling of different proportions of cell types. In this regard, it was challenging to assign TFs to cell type–specific processes given the mixed cell population and observational constraints of using human tissue. Changes in the relative proportions of cytotrophoblasts (decreasing) and STs (increasing) can be demonstrated histologically over the course of gestation. Collecting pure populations of trophoblasts could reduce the heterogeneity associated with bulk tissue samples that contain multiple cell types, though perhaps at the expense of losing information about the cell state in vivo.

In addition, we found that making comparisons between early versus late gestation placentas, and TSCs versus STs was not straightforward, limiting our ability to model molecular pathways from the placenta in cultured cells. In this regard, it is unclear why TFs with greater predicted activity in early pregnancy (i.e. MAFB, SNAI2, and ZBTB7C) would have similar or increased expression in STs compared to TSCs, although the effects could be explained by differences in TF activity or accessibility of binding sites. Finally, regarding the application of TFSEE, we note that TF mRNA levels may not accurately represent protein abundance or activity. As such, TFSEE may exclude TFs relevant to placenta biology under these circumstances.

## Supplementary Material

Owen_Placenta_Enh_Revised-Suppl_Figures_080223_ioad119

Supplementary_Table_ioad119

New_Microsoft_Word_Document_ioad119

## Data Availability

The genomic datasets generated for this study can be accessed from the NCBI’s Gene Expression Omnibus (GEO) repository (www.ncbi.nlm.nih.gov/geo/) using accession number GSE222035. All other data generated in this study are available within the article and its supplementary data files. The pipelines and scripts used for the computational analyses are available on GitHub at: https://github.com/Kraus-Lab/Transcriptome-changes-in-Placenta.
