# Supplementary material for: Genome-wide identification of transcriptional enhancers during human placental development and association with function, differentiation, and disease
Source: Biol Reprod. 2023 Sep 23;109(6):965–81. doi: 10.1093/biolre/ioad119 (PMC10724456; doi:10.1093/biolre/ioad119)
Supplement: Owen_Placenta_Enh_Revised-Suppl_Figures_080223_ioad119 [file owen_placenta_enh_revised-suppl_figures_080223_ioad119.pdf]

Supplementary Figures and Tables

"Genome-Wide Identification of Transcriptional Enhancers During Human Placental Development and Association with Function, Differentiation, and Disease"

Owen *et al.* (2023)

Supplementary Figures S1 through S4 *(see below)*
Supplementary Tables S1 through S10 *(see Excel files)*

This document contains the following supplementary figures and supplementary table legends:

Page

- **Supplementary Figure S1.** Expression levels by RNA-seq from bulk placenta tissue of genes enriched in single cell RNA-seq data sets....3
- **Supplementary Figure S2.** Comparisons of differentially regulated genes and enhancers between trimesters....4
- **Supplementary Figure S3.** Expression levels of key marker genes by RNA-seq and by RT-qPCR in TSCs and STs....5
- **Supplementary Figure S4.** Expression of marker genes upon knockdown of ZBTB7C or SNAI2 in undifferentiated TSCs....6
- **Supplementary Table S1.** Enhancer transcription for each placenta across the trimesters....7
- **Supplementary Table S2.** Gene expression for each placenta across the trimesters....7
- **Supplementary Table S3.** Complete gene ontology tables for sets of genes whose expression decreases (E) or increases (F) across gestation....7
- **Supplementary Table S4.** Complete gene ontology tables for pairwise analyses of gene expression that are upregulated between trimesters....7
- **Supplementary Table S5.** Enhancers enriched in each trimester and the nearest neighboring genes....7
- **Supplementary Table S6.** Gene ontology analysis for the nearest neighboring genes to the subset of 3,550 enhancers which do not overlap any ENCODE annotated cCREs....7

- **Supplementary Table S7.** Enhancer transcription for common genes that are upregulated from RNAseq data and nearest neighbor genes from enhancers that are upregulated between trimesters 7
- **Supplementary Table S8.** p-values for all the correlations between gene and enhancer expression for FLT1. The "Column Heading" key provides annotation information 7
- **Supplementary Table S9.** Overlap of the location of eQTLs for gene expression in placenta tissue with the enhancers we defined 7
- **Supplementary Table S10.** Linear modeling for changes in TFSEE score for each transcription factor by trimester 8

Supplementary Figures

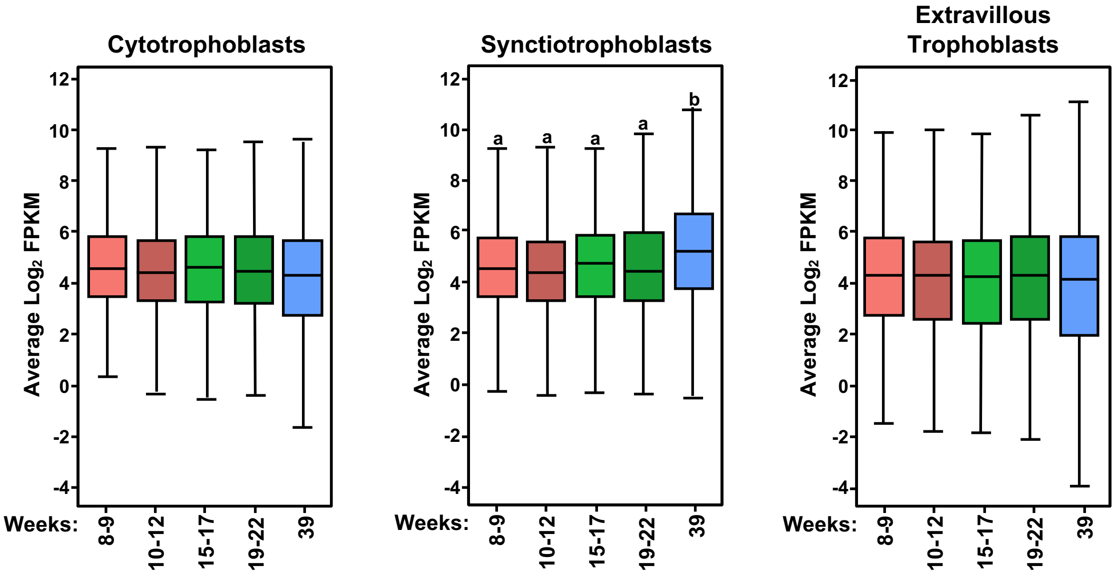

Supplementary Figure S1. Expression levels by RNA-seq from bulk placenta tissue of genes enriched in single cell RNA-seq data sets. Average expression of the gene signatures from the Human Protein Atlas database of cytotrophoblasts (420 genes), syncytiotrophoblasts (790 genes), and extravillous trophoblasts (680 genes). The data are segregated by the gestational ages indicated. The extravillous and cytotrophoblast signals do not significantly change between gestational age groups, while the syncytiotrophoblast signal is significantly increased in the 3rd trimester (bars marked with different letters, *a* and *b*, are significantly different; Wilcoxon rank-sum test with continuity correction, p-value = 1.904×10^{-7}).

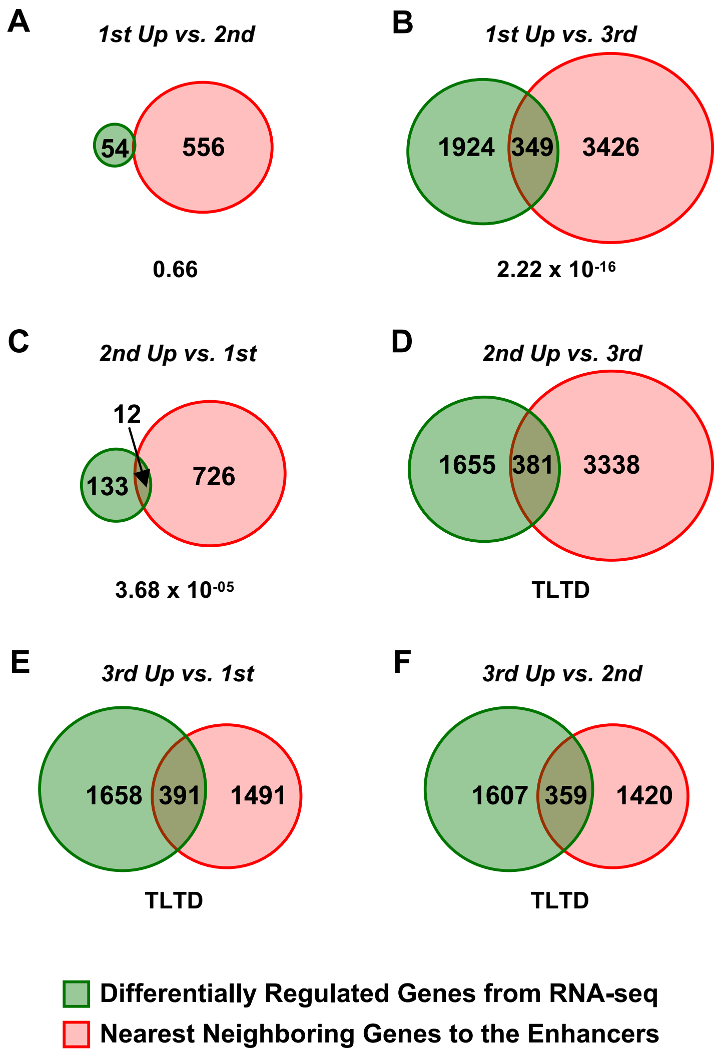

Supplementary Figure S2. Comparisons of differentially regulated genes and enhancers between trimesters. Venn diagrams showing the relationships between (1) the differentially regulated genes from RNA-seq *(green)* and (2) nearest neighbor genes for each identified enhancer *(red)* plotted for the trimester combinations shown. The numbers below the Venn diagrams indicate the probability of observing k = [n of overlap] or more genes common to both sets determined by a hypergeometric distribution test (i.e., when compared to the probability of overlap for randomly selected gene lists in sets of the same size).

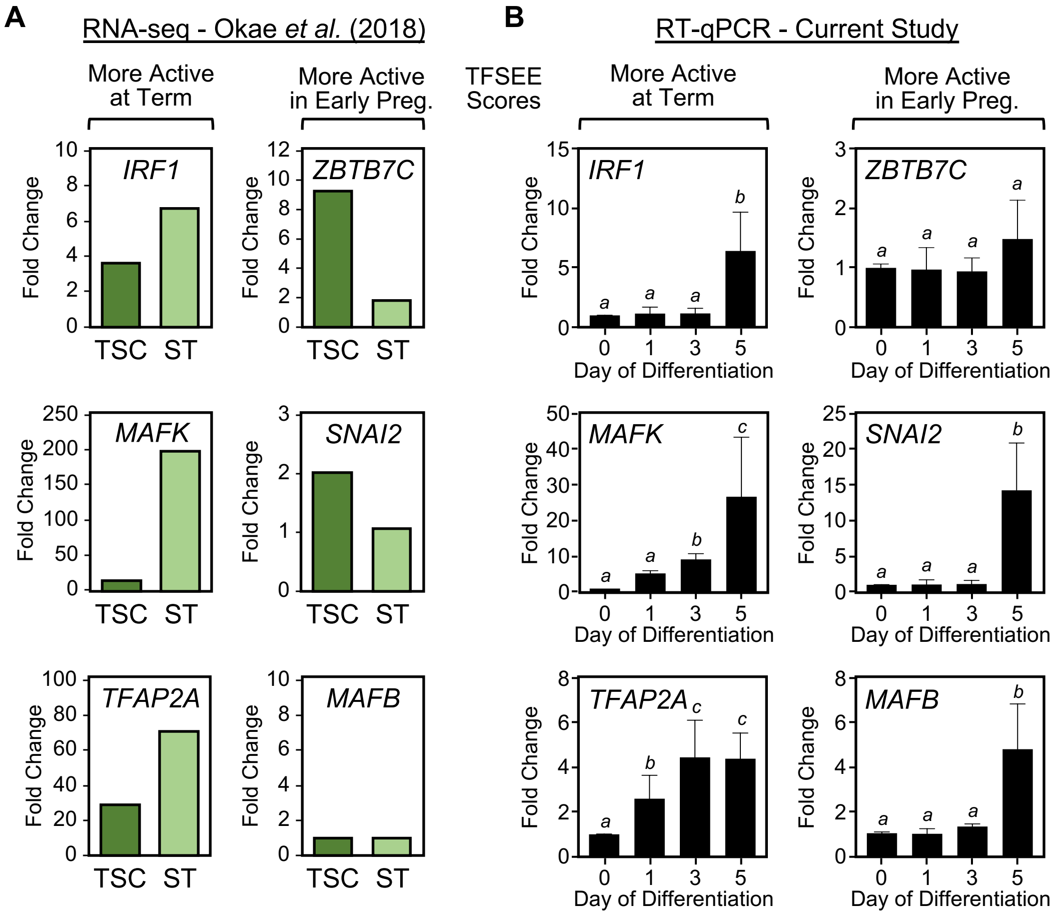

Supplementary Figure S3. Expression levels of key marker genes by RNA-seq and by RT-qPCR in TSCs and STs. The RNA-seq are from Okae *et al.* (2018) (TSCs versus STs) and the RT-qPCR data are from the current study (time course of differentiation of TSCs). For ease of comparison, the data are expressed as fold change and the genes are separated into their TFSEE groups ("active in early pregnancy" and "active at term" as indicated).

(A) Expression of the genes by RNA-seq.

(B) For the RT-qPCR analyses, TSCs were differentiated into STs over a 5 day time course. RNA was isolated from the cells and subjected to RT-qPCR analysis. Significance was determined by ANOVA. Bars marked with different letters (*a, b, c*) are significantly different, $p < 0.05$

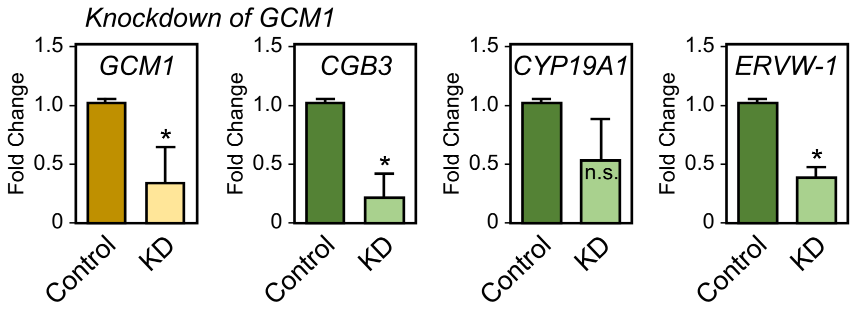

Supplementary Figure S4. Expression of marker genes upon knockdown of *GCM1* in TSCs. Undifferentiated TSCs were subjected to siRNA-mediated knockdown of *GCM1* and then subjected to Taqman RT-qPCR analysis. Knockdown was confirmed by the expression of *GCM1*. Effects of *GCM1* knockdown on the expression of the marker genes shown were also determined. Significance was determined by Student's t-test; * = $p < 0.05$, n.s. = not significant.

Supplementary Table Legends

Supplementary Table S1. Enhancer transcription for each placenta across the trimesters. The "Column Heading" key provides annotation information. The "Tab" key provides details about all the other worksheets contained within this spreadsheet.

Supplementary Table S2. Gene expression for each placenta across the trimesters. The "Tab" key provides details about all the other worksheets contained within this spreadsheet.

Supplementary Table S3. Complete gene ontology tables for sets of genes whose expression decreases (E) or increases (F) across gestation. Panther Database was used to curate the gene ontologies for sets of genes whose expression decreases or increases across gestation. The "Column Heading" key provides annotation information. The "Tab" key provides details about all the other worksheets contained within this spreadsheet.

Supplementary Table S4. Complete gene ontology tables for pairwise analyses of gene expression that are upregulated between trimesters. Panther Database was used to curate the gene ontologies for sets of genes whose expression are upregulated between trimesters. The "Column Heading" key provides annotation information. The "Tab" key provides details about all the other worksheets contained within this spreadsheet.

Supplementary Table S5. Enhancers enriched in each trimester and the nearest neighboring genes. The "Column Heading" key provides annotation information. The "Tab" key provides details about all the other worksheets contained within this spreadsheet.

Supplementary Table S6. Gene ontology analysis for the nearest neighboring genes to the subset of 3,550 enhancers which do not overlap any ENCODE annotated cCREs. GREAT Database was used to curate the gene ontologies for sets of genes whose expression are upregulated between trimesters. The "Column Heading" key provides annotation information. The "Tab" key provides details about all the other worksheets contained within this spreadsheet.

Supplementary Table S7. Enhancer transcription for common genes that are upregulated from RNAseq data and nearest neighbor genes from enhancers that are upregulated between trimesters. The "Column Heading" key provides annotation information. The "Tab" key provides details about all the other worksheets contained within this spreadsheet.

Supplementary Table S8. p-values for all the correlations between gene and enhancer expression for *FLT1*. The "Column Heading" key provides annotation information. The "Tab" key provides details about all the other worksheets contained within this spreadsheet.

Supplementary Table S9. Overlap of the location of eQTLs for gene expression in placenta tissue with the enhancers we defined. 1,216 eQTLs from extragenic regions of the genome reported by Peng *et al.* (2017) with the enhancers we defined, 80 of these map to enhancer regions identified in this study. The "Column Heading" key provides annotation information. The "Tab" key provides details about all the other worksheets contained within this spreadsheet.

Supplementary Table S10. Linear modeling for changes in TFSEE score for each transcription factor by trimester. Of the 101 transcription factors identified in the TFSEE analysis, 30 showed a statistically significant trend across gestation by trimester. While Fig. 5C is based on the 36 TFs identified through clustering analysis, we note that 26 of these are also statistically significant in the linear modeling analysis. The "Column Heading" key provides annotation information. The "Tab" key provides details about all the other worksheets contained within this spreadsheet.
