# Supplementary material for: Genome-wide identification of transcriptional enhancers during human placental development and association with function, differentiation, and disease
Source: Biol Reprod. 2023 Sep 23;109(6):965–81. doi: 10.1093/biolre/ioad119 (PMC10724456; doi:10.1093/biolre/ioad119)
Supplement: New_Microsoft_Word_Document_ioad119 [file new_microsoft_word_document_ioad119.docx]

**Supplementary Data Files**

This paper contains the following supplementary data files:

• One Supplemental Figure File (.pdf), containing:

- Supplementary Figure S1. Expression levels by RNA-seq from bulk placenta tissue of genes enriched in single cell RNA-seq data sets.

- Supplementary Figure S2. Comparisons of differentially regulated genes and enhancers between trimesters.

- Supplementary Figure S3. Expression levels of key marker genes by RNA-seq and by RT-qPCR in TSCs and STs.

- Supplementary Figure S4. Expression of marker genes upon knockdown of ZBTB7C or SNAI2 in undifferentiated TSCs.

• Ten Supplemental Table Files (.xlsx or xlsb), containing:

- Supplementary Table S1. Enhancer transcription for each placenta across the trimesters.

- Supplementary Table S2. Gene expression for each placenta across the trimesters.

- Supplementary Table S3. Complete gene ontology tables for sets of genes whose expression decreases (E) or increases (F) across gestation.

- Supplementary Table S4. Complete gene ontology tables for pairwise analyses of gene expression that are upregulated between trimesters.

- Supplementary Table S5. Enhancers enriched in each trimester and the nearest neighboring genes.

- Supplementary Table S6. Gene ontology analysis for the nearest neighboring genes to the subset of 3,550 enhancers which do not overlap any ENCODE annotated cCREs.

- Supplementary Table S7. Enhancer transcription for common genes that are upregulated from RNAseq data and nearest neighbor genes from enhancers that are upregulated between trimesters.

- Supplementary Table S8. p-values for all the correlations between gene and enhancer expression for FLT1. The "Column Heading" key provides annotation information.

- Supplementary Table S9. Overlap of the location of eQTLs for gene expression in placenta tissue with the enhancers we defined.

- Supplementary Table S10. Linear modeling for changes in TFSEE score for each transcription factor by trimester.
